# Handgrip strength and diameter–product–derived psoas area estimate in end–stage liver cirrhosis: an exploratory single–center analysis

**DOI:** 10.1007/s00423-026-04150-y

**Published:** 2026-07-29

**Authors:** Eugen Malamutmann, Christina Neuhaus, Lale Umutlu, Sophia T. Schmitz, Miroslav Jalc, Christian G. Klein, Jan Bednarsch, Ulf Neumann, Arzu Oezcelik

**Affiliations:** 1 Department of General, Visceral, Vascular and Transplantation Surgery, University Medicine Essen, University of Duisburg-Essen, Hufelandstraße 55, Essen, 45147 Germany; 2 Institute of Diagnostic and Interventional Radiology and Neuroradiology, University Medicine Essen, University of Duisburg-Essen, Essen, Germany; 3 Institute of Sex and Gender Sensitive Medicine, University Medicine Essen, University of Duisburg-Essen, Essen, Germany; 4 Department of Surgery, Maastricht University Medical Center, Maastricht, The Netherlands

**Keywords:** Sarcopenia, Liver cirrhosis, Handgrip strength, Psoas muscle, Liver transplantation, MELD score, Frailty

## Abstract

**Background:**

Previous studies suggest that handgrip strength may correlate with muscle mass in cirrhosis, but validation across diverse transplant cohorts remains limited. We sought to validate whether previously reported correlations between handgrip strength and computed tomography (CT)-derived psoas-area estimates hold in a single-center cohort of liver transplant candidates and to explore whether a bedside prediction model could be derived.

**Methods:**

In this retrospective cohort study, 96 patients with end-stage liver cirrhosis undergoing transplant evaluation at University Hospital Essen (January 2020–December 2024) underwent standardized handgrip strength, 4-meter gait time, and diameter-product-derived psoas area estimates. Pearson correlation, multiple linear regression, and Cox proportional hazards analyses were performed with Benjamini–Hochberg correction for multiple comparisons.

**Results:**

Handgrip strength significantly correlated with total psoas area (*r = 0.35*, 95% CI 0.16–0.51, $p_\textrm{adj} = 0.002$). Multiple linear regression yielded a prediction model ($R^2 = 0.27$) achieving 72% sensitivity and 68% specificity for sarcopenia detection (AUC = 0.74). In multivariate Cox regression, each 100 mm^2^/m^2^ increase in psoas index reduced waiting list mortality by 15% (HR = 0.85, 95% CI 0.73–0.98, *p = 0.032*) and post-transplant mortality by 18% (HR = 0.82, 95% CI 0.71–0.95, *p = 0.002*).

**Conclusion:**

Handgrip strength shows a modest, statistically significant association with diameter-product-derived psoas area estimates in liver transplant candidates. The derived prediction model has limited accuracy ($R^2 = 0.27$, AUC = 0.74) and cannot defer clinically indicated CT imaging; it may assist in identifying candidates who warrant confirmatory imaging or closer nutritional follow-up.

**Supplementary Information:**

The online version contains supplementary material available at 10.1007/s00423-026-04150-y.

## Introduction

Liver transplantation remains the definitive therapy for end-stage liver cirrhosis. With persistent organ shortages and waiting list mortality, optimizing patient selection has become paramount [1]. The Model for End-Stage Liver Disease (MELD) score provides objective disease severity assessment but has recognized limitations in sarcopenic patients whose low creatinine may underestimate disease severity [2].

Sarcopenia, the progressive loss of skeletal muscle mass and function [3], has emerged as a mortality predictor in liver cirrhosis [4]. The European Working Group on Sarcopenia in Older People (EWGSOP2) emphasizes impaired muscle strength as the primary diagnostic characteristic, with detection of low muscle quantity and quality confirming diagnosis [3]. In cirrhotic patients, sarcopenia prevalence ranges from 17% to 49% depending on assessment method [5].

Computed tomography-based measurement of skeletal muscle at the L3 level represents the reference standard for quantifying muscle mass in cirrhotic patients [6]. The psoas muscle index, normalized for height squared, has demonstrated predictive value for waiting list mortality [7]. However, CT imaging requires radiation, specialized software, and is not readily available for routine monitoring.

Qualitative assessments including handgrip strength and 4-meter gait time offer practical bedside alternatives. Handgrip strength independently predicts mortality in cirrhosis [8, 9], and recent meta-analyses confirm that handgrip-MELD combinations outperform imaging-based sarcopenia measures for mortality prediction [10, 11]. Despite the utility of both approaches, the quantitative relationship between functional and morphometric sarcopenia measures in end-stage liver cirrhosis remains incompletely characterized.

Based on prior studies suggesting associations between functional and morphometric sarcopenia markers, we sought to validate these findings in our single-center transplant cohort. The primary objective was to test whether previously reported correlations between handgrip strength and psoas-area estimates hold in German liver transplant candidates. Secondary objectives included evaluating whether a simple prediction model could be derived and confirming the prognostic significance of psoas index for waiting list and post-transplant survival.

## Materials and methods

This retrospective cohort study was conducted at the Department of General, Visceral, Vascular and Transplantation Surgery, University Hospital Essen, Germany. All adult patients (*\ge *18 years) with end-stage liver cirrhosis presenting for initial transplant evaluation between January 1, 2020, and December 31, 2024, were eligible. Inclusion criteria required: (1) confirmed end-stage liver cirrhosis; (2) documented handgrip strength and 4-meter gait time measurements; and (3) abdominal CT imaging within ±90 days of evaluation. Patients with Grade 3–4 hepatic encephalopathy precluding reliable functional assessment (*n = 3*) were excluded. Complete case analysis was performed; patients with missing measurements were excluded. The study was approved by the Ethics Committee of the University of Duisburg-Essen (file number 20-9765-BO) and conducted in accordance with the 1964 Declaration of Helsinki and its later amendments. Due to the retrospective nature, informed consent was waived. All patients subsequently undergoing transplantation received deceased donor liver transplantation in standard technique with bicaval anastomosis. Intraoperative parameters including packed red blood cell transfusion, cold ischemia time, and warm ischemia time were recorded. Postoperative complications were graded according to the Clavien–Dindo classification [12].

For the primary correlation analysis, a sample of *n = 96* provided >90% power to detect a correlation of *r = 0.35* at *α = 0.05* (two-tailed). For multiple regression with three predictors, this sample exceeds the minimum of 10–15 observations per predictor. However, survival analyses with 13 events per outcome should be considered exploratory given limited events-per-variable ratios.

Handgrip strength was measured using a calibrated hydraulic hand dynamometer (SAEHAN Corporation, South Korea; calibrated monthly). Patients were seated with shoulder adducted, elbow flexed at 90$^\circ$, forearm neutral, and wrist in 0–30$^\circ$ dorsiflexion. Upon command, patients performed three maximum-effort contractions with the dominant hand. Mean values were recorded in kilograms.

Patients walked a marked 4-meter distance at their “normal comfortable pace”; walking aids were permitted. Time was recorded from start command until reaching the endpoint. The test was repeated and the mean time calculated. We report this metric as *4-meter gait time (seconds)*; longer time indicates poorer performance and a positive Cox HR per second therefore corresponds to higher mortality risk.

Psoas muscle area was measured on abdominal CT scans (Siemens SOMATOM, 5 mm slice thickness) using institutional PACS (Centricity Enterprise Web). Measurements were performed at L3/4 level: for each side, the largest longitudinal diameter was multiplied by the largest transverse diameter. Total psoas area was the sum of right and left measurements (mm^2^). The psoas index was calculated as total psoas area divided by height squared (mm^2^/m^2^). This diameter-product approach yields an *estimate* of psoas area; it is not a planimetric cross-sectional tracing and systematically over-estimates true planimetric psoas cross-sectional area for any non-rectangular muscle profile. Values reported here are therefore not directly interchangeable with planimetric L3-based skeletal-muscle indices used in published thresholds.

Twenty randomly selected scans were independently measured by two radiologists blinded to clinical data. Inter-observer reliability for total psoas area demonstrated excellent agreement (intraclass correlation coefficient = 0.92, 95% CI 0.82–0.97).

Demographics, pre-, intra- and postoperative data including anthropometrics, MELD score, grade of ascites (none/Grade 1 vs. Grade 2–3), encephalopathy, co-morbidities, donor data, time of surgery, cold and warm ischemia time, blood transfusion, ICU and hospital stay were documented. Follow-up including waiting list status, transplantation, and survival was collected through December 31, 2025.

Analyses were performed using DATAtab (DATAtab e.U., Graz, Austria). Continuous variables were expressed as mean ± SD and median with range. Pearson correlation coefficients were calculated with Benjamini–Hochberg false discovery rate (FDR) correction for multiple comparisons. Simple and multiple linear regression developed prediction models.

Discrimination of low diameter-product-derived psoas index was evaluated using sex-specific thresholds adapted from Carey et al. [13] (males: <545 mm^2^/m^2^; females: <385 mm^2^/m^2^). These thresholds were obtained by applying a study-specific 10% adjustment to the published L3 cutoffs to account for the L3/4 measurement level used here; this adjustment is an operational assumption and has not been independently validated. Diagnostic-accuracy outputs (sensitivity, specificity, predictive values, AUC) reported below are therefore exploratory and refer to discrimination of a low *diameter-product-derived* psoas index, not to validated whole-body sarcopenia detection.

Survival analysis employed Kaplan–Meier curves with log-rank tests and Cox proportional hazards regression. Hazard ratios for psoas index were reported per 100 mm^2^/m^2^ increment for clinical interpretability. A two-sided *p < 0.05* was considered significant.

## Results

Ninety-six patients with end-stage liver cirrhosis comprised the study cohort: 55 males (57.3%) and 41 females (42.7%). Mean age was 51.6 ± 11.4 years (range 20–69). Mean MELD score was 12.9 ± 4.6 (range 0–25). Clinically significant ascites (Grade 2–3) was present in 40 patients (41.7%). Complete baseline characteristics are presented in Table 1.

Mean handgrip strength was 40.9 ± 19.9 kg (range 5–115). Mean diameter-product-derived total psoas area estimate was 2822 ± 992 mm^2^ (range 1444–5482), with mean psoas index of 950 ± 299 mm^2^/m^2^ (range 446–1719).

Males demonstrated significantly greater handgrip strength (47.7 vs. 31.9 kg, *p < 0.001*), psoas area estimate (3265 vs. 2228 mm^2^, *p < 0.001*), and psoas index (1075 vs. 783 mm^2^/m^2^, *p < 0.001*) compared to females (Online Resource 1). BMI, MELD score, and gait time did not differ significantly between sexes.

**Fig. 1:**
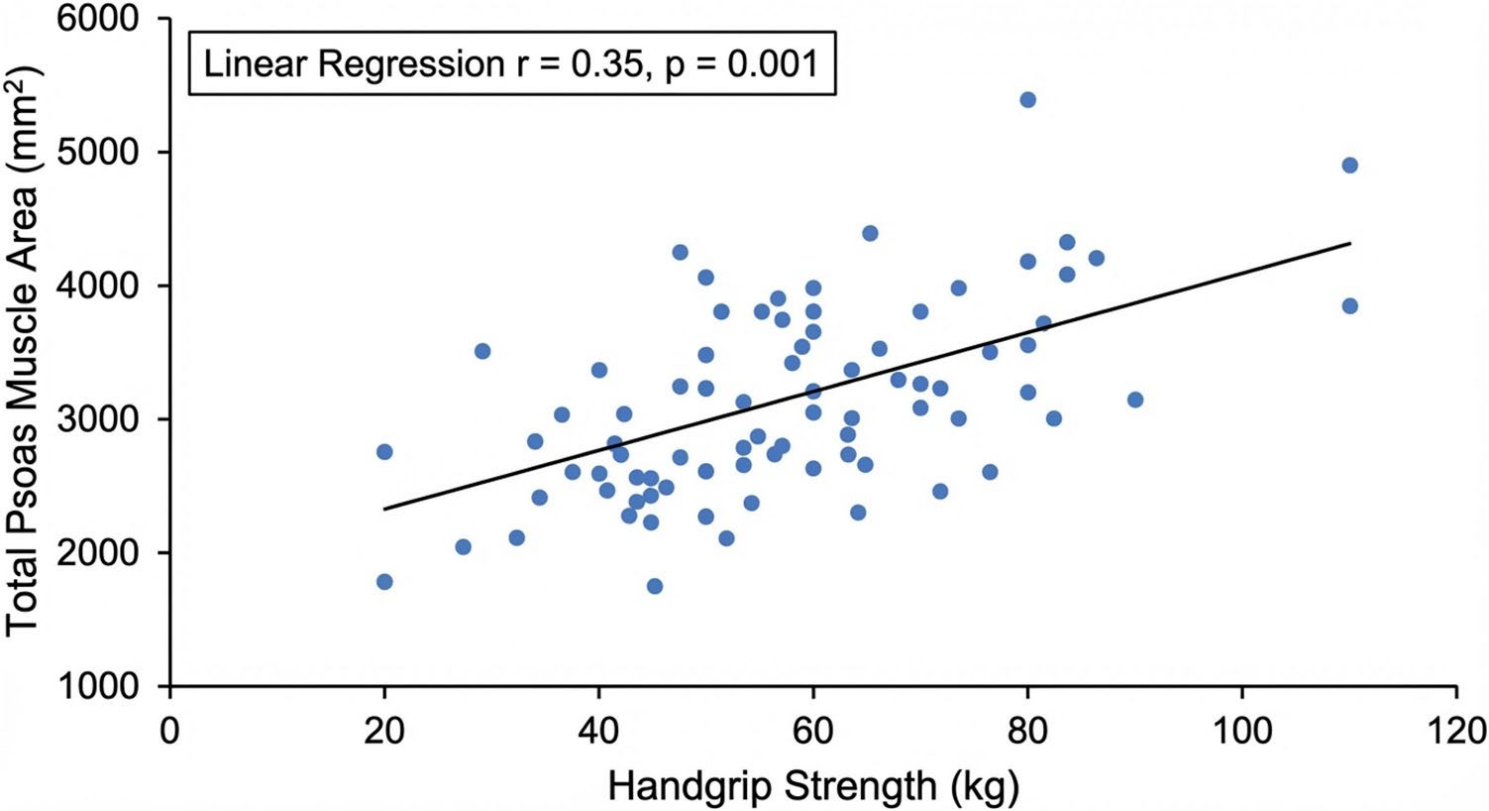
Correlation between handgrip strength and the diameter-product-derived psoas area estimate. Scatter plot demonstrating significant positive correlation (*r = 0.35*, 95% CI 0.16–0.51, $p_\textrm{adj} = 0.002$) between handgrip strength (kg) and diameter-product-derived total psoas area estimate (mm^2^) in 96 patients with end-stage liver cirrhosis

Handgrip strength demonstrated a significant moderate positive correlation with the psoas area estimate (*r = 0.35*, 95% CI 0.16–0.51, $p_\textrm{adj} = 0.002$; Fig. 1) and psoas index (*r = 0.30*, $p_\textrm{adj} = 0.008$). Gait time showed no significant correlation with psoas area (*r = -0.19*, *p = 0.064*) or psoas index (*r = -0.17*, *p = 0.100*). Body weight (*r = 0.38*, $p_\textrm{adj} = 0.002$) and height (*r = 0.42*, $p_\textrm{adj} < 0.001$) also correlated significantly with the psoas area estimate. MELD score showed no correlation with psoas measurements (*r = -0.03*, *p = 0.790*). Complete correlation data are presented in Online Resource 2.

Simple linear regression of handgrip strength on the psoas area estimate yielded: Total Psoas Area Estimate = 2111.06 + 17.4 *× * Handgrip Strength ($R^2 = 0.12$, SEE = 935.21 mm^2^).

Multiple linear regression incorporating handgrip strength, weight, and height produced an improved model (Online Resource 3; Online Resource 4):

1 $$\begin{aligned} \text {Total Psoas Area} = -3405.99 \\+ 14.25 \times \text {Weight} + 26.64 \\\times \text {Height} + 12.12 \times \text {Handgrip} \end{aligned}$$

This model explained 27% of variance ($R^2 = 0.27$, SEE = 858.65 mm^2^). Standardized coefficients indicated approximately equal contributions from weight (*β = 0.25*), height (*β = 0.23*), and handgrip (*β = 0.24*).

Using sex-specific psoas index thresholds adapted for L3/4 measurements, 31 patients (32.3%) met criteria for sarcopenia. The prediction model achieved sensitivity of 72% (95% CI 53–87%), specificity of 68% (95% CI 56–79%), PPV of 52%, NPV of 83%, and AUC of 0.74 (95% CI 0.64–0.84) for sarcopenia detection.

Among patients without clinically significant ascites (*n = 56*), the handgrip-psoas correlation remained significant (*r = 0.38*, *p = 0.004*). In patients with Grade 2–3 ascites (*n = 40*), the correlation was attenuated but remained significant (*r = 0.29*, *p = 0.042*). Model performance was similar in both subgroups ($R^2 = 0.29$ vs. 0.24).

During follow-up, 13 patients (13.5%) died while awaiting transplantation (mean waiting time: 43.1 ± 18.4 months). Fifty-three patients (55.2%) underwent transplantation, of whom 13 (24.5%) died during follow-up (mean post-transplant survival: 38.6 ± 19.1 months).

In univariate analysis, male sex (log-rank *p = 0.022*; Online Resource 5), MELD score (*p = 0.015*), psoas area (*p = 0.018*), and psoas index (*p = 0.020*) were associated with waiting list survival. Multivariate Cox regression identified three predictors that retained statistical significance in the exploratory multivariable model (Table 2): MELD score (HR = 1.20 per point, 95% CI 1.05–1.39, *p = 0.009*), gait time (HR = 1.02 per second of 4-meter walk, 95% CI 1.00–1.04, *p = 0.041*), and psoas index (HR = 0.85 per 100 mm^2^/m^2^, 95% CI 0.73–0.98, *p = 0.032*).

**Fig. 2:**
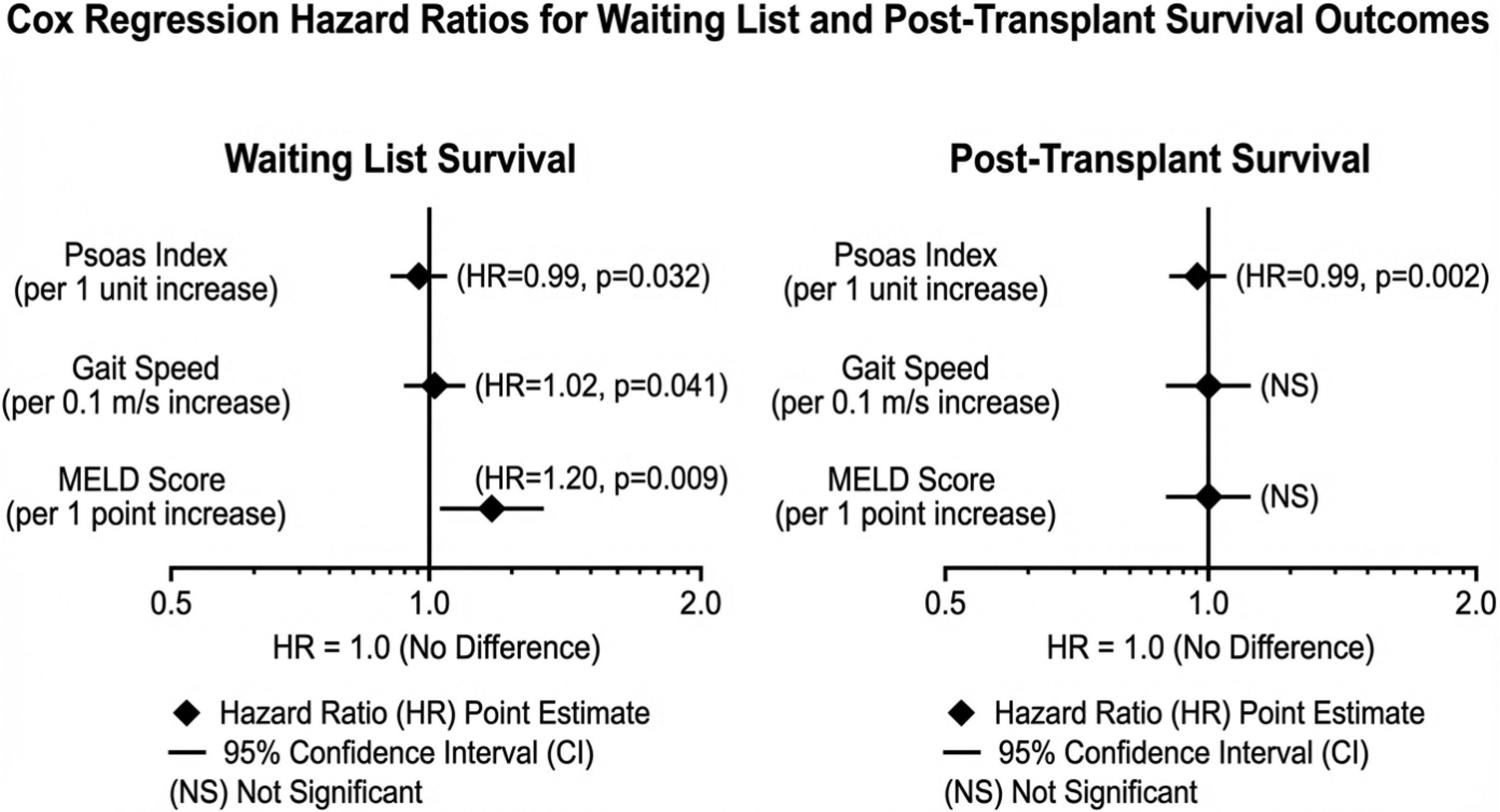
Forest plot of Cox regression hazard ratios. Multivariate hazard ratios with 95% confidence intervals. Psoas index reported per 100 mm^2^/m^2^; MELD per point; gait time per second. HR < 1 indicates protective effect

Among transplanted patients (*n = 53*), univariate analysis identified age (*p = 0.039*) and psoas index (*p = 0.002*) as significant predictors. In multivariate analysis, only psoas index retained significance in the exploratory multivariable model (HR = 0.82 per 100 mm^2^/m^2^, 95% CI 0.71–0.95, *p = 0.002*). Neither gait time nor MELD score predicted post-transplant survival (Table 2; Fig. 2).

The survival analyses revealed stage-specific prognostic patterns. During the waiting list period, MELD score, gait time, and psoas index retained significance in the exploratory multivariable Cox model. Post-transplantation, only psoas index retained prognostic significance, while gait time and MELD failed to predict outcomes.

**Table 1:** Baseline characteristics of the study population (*n = 96*)

Parameter	Mean ± SD	Median	Range
Age (years)	51.6 ± 11.4	54	20–69
Weight (kg)	81.1 ± 17.3	79	45–125
Height (cm)	171.8 ± 8.5	172	150–192
BMI (kg/m^2^)	27.4 ± 5.3	26	17–44.3
MELD score	12.9 ± 4.6	12	0–25
Handgrip strength (kg)	40.9 ± 19.9	40	5–115
Gait time (seconds, 4-meter walk)^a^	8.4 ± 19.1	4	2–99
Total psoas area estimate (mm^2^)	2822 ± 992	2490	1444–5482
Total psoas index (mm^2^/m^2^)	950 ± 299	880	446–1719
Categorical variables, *n* (%)			
Male sex	55 (57.3%)		
Female sex	41 (42.7%)		
Ascites Grade 2–3	40 (41.7%)		

^a^Maximum 99 seconds reflects one patient with severe deconditioning requiring multiple rest stops; sensitivity analysis excluding this outlier did not change results

## Discussion

Sarcopenia is a highly prevalent and clinically consequential complication of liver cirrhosis, reflecting the profound metabolic, inflammatory, and hormonal derangements that accompany advanced chronic liver disease. Its presence is independently associated with increased morbidity, waitlist mortality, and adverse post-transplant outcomes, underscoring its importance as a key determinant of prognosis in cirrhosis.

However, several diagnostic options for assessment of muscle quality and quantity exist and evidence on comparability is scarce.

The present single-center validation investigation has yielded findings that appear to corroborate a statistically significant association between handgrip dynamometric strength and computed tomography-derived psoas muscle area estimate (*r = 0.35*, $p_\textrm{adj} = 0.002$) in patients presenting with end-stage cirrhosis who were undergoing evaluation for liver transplantation. This is consistent with previously published reports from geographically and demographically distinct transplant populations. These observations may be interpreted as providing supplementary evidence supporting the proposition that bedside functional assessment methodologies possess correlative validity with quantitative morphometric muscle mass determinations. However, there are certain limitations that warrant careful consideration in clinical implementation.

The magnitude of the observed correlation coefficient demonstrates substantial alignment with the extant body of literature pertaining to functional-morphometric associations in chronic hepatopathy. Specifically, the investigation conducted by Wang and colleagues documented a comparable correlation between handgrip dynamometry and skeletal muscle index quantification in cirrhotic populations (*r = 0.31*) [14], and the analyses performed by Tandon et al. revealed similar patterns (*r = 0.29*) in patients diagnosed with compensated hepatic cirrhosis [15].

The coefficient of determination derived from the multivariable prediction model ($R^2 = 0.27$) may be interpreted as accounting for approximately one-quarter of the observed variance in the psoas area estimate. Published correlational coefficients between handgrip strength determinations and various muscle mass quantification methodologies in hepatopathic cohorts have been documented to range from *r = 0.26* to *r = 0.58* [16, 17], with the presently observed *r = 0.35* falling squarely within this anticipated distribution, thereby suggesting that the present findings are neither anomalously elevated nor unexpectedly attenuated relative to established norms. Correspondingly, $R^2$ values ranging from 0.10 to 0.30 appear to represent the standard range for clinical screening models endeavoring to prognosticate muscle mass from functional assessments [18, 19], a phenomenon that may be attributable to the fundamental distinction between the physiological constructs captured by neuromuscular functional capacity versus morphometric tissue quantity determinations.

A recently conducted systematic literature review encompassing publications from 2020 through 2025 provides additional contextual framework for the interpretation of the present findings. In a Turkish multicenter cohort comprising 100 cirrhotic patients, handgrip strength demonstrated correlative associations with muscle mass indicators at *r = -0.49* (*p < 0.001*), explaining approximately 24% of variance [20], a magnitude that appears comparable to the 27% observed in the present investigation. It is noteworthy that the $R^2 = 0.27$ documented herein exceeds the typical literature values of 12–24%, a finding which may suggest that the incorporation of handgrip strength in conjunction with anthropometric covariables (weight, height) potentially captures supplementary predictive information not attainable through univariate modeling approaches. Furthermore, a 2025 Brazilian multicenter investigation (*n = 724*) established sex- and age-stratified handgrip strength cutoff values for prognosis of 1-year mortality in cirrhosis (*łe *33 kgf for males <60 years; *łe *22 kgf for males *\ge *60 years), with diminished handgrip strength conferring an approximately 2.5-fold elevation in mortality risk [21]. Moreover, Sinclair and colleagues demonstrated that the combination of handgrip strength with MELD scoring significantly outperformed the pairing of CT-measured muscle mass with MELD for the prediction of waiting list mortality (*p < 0.001*) [8], thereby reinforcing the potential clinical utility of functional assessment methodologies when employed alongside morphometric evaluation. These contemporary findings are supportive of the present validation results and appear to underscore the proposition that functional measures capture prognostically relevant information that is distinct from, and potentially complementary to, imaging-based muscle quantification approaches.

It is of considerable importance to acknowledge that the intended purpose of such predictive models is clinical screening rather than definitive diagnostic ascertainment. For screening applications, moderate discriminative capacity may be considered acceptable insofar as the primary objective pertains to patient triage rather than precise quantification. The present model achieved an area under the receiver operating characteristic curve of 0.74 (95% CI 0.64–0.84), a value which compares favorably with previously published sarcopenia screening instruments in cirrhosis that have characteristically demonstrated AUC values ranging from 0.70 to 0.85 [13, 22]. The sensitivity of 72% and negative predictive value of 83% reflect *moderate* discrimination of low diameter-product-derived psoas index and are not sufficient to defer clinically indicated CT imaging. The 28% false-negative rate confirms that diameter-product-derived psoas estimation cannot serve as a stand-alone screen and must be interpreted alongside cross-sectional imaging.

The absence of statistically significant correlation between gait time and psoas measurements constitutes an unexpected finding that merits careful consideration. Gait performance is understood to depend upon multiple factors extending beyond muscle mass per se, including neurological function, balance, cardiopulmonary reserve, and the potential confounding influence of hepatic encephalopathy. Notwithstanding the observed lack of correlation with morphometric muscle mass, gait time retained significance in the exploratory multivariable model for waiting-list mortality, a finding which may suggest that this functional parameter captures prognostic information that is distinct from morphometric sarcopenia indicators. This apparent dissociation between functional and morphometric parameters appears to provide support for the EWGSOP2 conceptual framework, which distinguishes muscle strength, quantity, and physical performance as complementary yet distinct domains requiring independent assessment [3].

The survival analyses undertaken in the present investigation identified psoas index as the sole predictor maintaining prognostic significance across both the waiting list and post-transplantation observation periods. Each 100 mm^2^/m^2^ increment in psoas index was associated with an approximate 15% reduction in waiting list mortality risk and an 18% reduction in post-transplant mortality risk. These effect size estimates appear to demonstrate alignment with meta-analytic estimates documenting sarcopenia-associated hazard ratios ranging from 1.55 to 1.84 [10, 11]. It is noteworthy that while MELD score and gait time demonstrated predictive capacity for waiting list outcomes, neither parameter maintained statistically significant prognostic value in the post-transplantation period, an observation which may be interpreted as underscoring sarcopenia’s unique prognostic relevance throughout the transplantation continuum.

The potentially confounding influence of ascites on body weight measurement was addressed through sensitivity analysis. The handgrip-psoas correlation remained statistically significant regardless of ascites status, albeit with slight attenuation observed in patients presenting with Grade 2–3 ascites (*r = 0.29* versus 0.38). This apparent robustness may suggest that the model retains clinical utility across the ascites severity spectrum, though it should be noted that dry weight estimation, when feasible, might potentially improve predictive accuracy.

Several methodological limitations warrant acknowledgment. First, the retrospective single-center design constrains generalizability. Second, the survival analyses, predicated on 13 events per outcome, are exploratory; the events-per-variable ratio falls below the recommended 10:1 threshold for stable Cox regression modeling, and external validation in independent cohorts is required before clinical implementation. Third, the cohort had relatively modest disease severity (mean MELD 12.9 ± 4.6, range 0–25), reflecting a transplant-evaluation population that includes well-compensated candidates; this distribution may have contributed to attenuation of the prognostic signal of bedside functional measures, since handgrip’s prognostic strength has been most pronounced in cohorts with more advanced disease [8, 15]. Fourth, the diameter-product method employed at the L3/4 level yields an *estimate* of psoas area rather than a planimetric cross-sectional measurement, and psoas area itself — regardless of slice level or measurement technique — represents an anatomically restricted proxy for global skeletal muscle mass; the L3 skeletal-muscle index (L3-SMI), computed by planimetric tracing of all muscles at L3, remains the recognized reference standard [6, 13]. Whole-skeletal-muscle segmentation was not generated for the present cohort and is identified as a priority for follow-up work. Fifth, the ±90-day CT timing window introduces potential measurement variability that may have attenuated observed associations. Sixth, the cross-sectional design with single-time-point handgrip and CT measurements precludes assessment of muscle-mass trajectory or training response, particularly relevant in transplant candidates whose clinical status evolves over the waiting period. Seventh, the moderate $R^2$ indicates substantial unexplained variance, likely reflecting unmeasured determinants of psoas area — including nutritional status, systemic inflammation, physical activity, and disease duration — that were not systematically captured in analyzable form for this retrospective cohort. Eighth, sex-stratified correlation analyses were not performed; given significant inter-sex differences in handgrip and psoas measurements, separate analyses may reveal gender-specific associations.

Several future directions emerge from these limitations. First, prospective replication should incorporate L3 planimetric skeletal-muscle index (L3-SMI) alongside diameter-product-derived psoas estimates, ideally with automated segmentation pipelines, to quantify the partial-proxy gap and to align thresholds with the published reference standard. Second, longitudinal repeat measurements during the waiting list (e.g., 3-month intervals) and post-transplant (6 and 12 months) would permit assessment of dynamic muscle-mass trajectories and training response, which the present cross-sectional design cannot address. Third, prospective inclusion of nutritional status (e.g., dietary recall, prealbumin), systemic inflammation (CRP, IL-6), physical activity, and precise disease duration in multivariable models could substantially improve explanatory power. Fourth, volumetric whole-body skeletal muscle analysis may yield stronger correlations with functional measures than single-slice approaches and could be integrated with functional measures for combined screening. Empirical verification of these hypotheses requires multicenter prospective studies with standardized protocols.

**Table 2:** Cox proportional hazards regression for survival outcomes^a^

Variable	HR	95% CI	*p*-value
*Waiting list survival (multivariate)*			
MELD score (per point)	1.20	1.05–1.39	0.009
Gait speed (per second)	1.02	1.00–1.04	0.041
Psoas index (per 100 mm^2^/m^2^)	0.85	0.73–0.98	0.032
*Post-transplant survival (multivariate)*			
Psoas index (per 100 mm^2^/m^2^)	0.82	0.71–0.95	0.002
Gait speed (per second)	0.93	0.67–1.29	0.664
MELD score (per point)	0.99	0.88–1.12	0.895

^a^Hazard ratios for psoas index reported per 100 mm^2^/m^2^ for clinical interpretability

Waiting list analysis: *n = 13* events; Post-transplant analysis: *n = 13* events

## Conclusion

This single-center exploratory analysis is consistent with previously reported associations between handgrip strength and psoas-area estimates in liver transplant candidates. The observed correlation (*r = 0.35*) falls within the published range for functional–morphometric correlations in cirrhosis, supporting an association but not establishing handgrip strength as a substitute for cross-sectional imaging. The derived prediction model ($R^2 = 0.27$, AUC = 0.74) has only moderate discrimination of low diameter-product-derived psoas index and is best interpreted as complementary to CT-based assessment rather than a screening replacement. Our exploratory survival analyses, predicated on 13 events per outcome, are hypothesis-generating and must be confirmed in larger multicenter cohorts before clinical implementation. Overall, this analysis contributes incremental evidence on bedside functional measures in liver transplant evaluation, while underscoring the need for L3-based whole-skeletal-muscle quantification, longitudinal repeat measurements, and external validation.

## Supplementary Information

Below is the link to the electronic supplementary material.

Supplementary file 1 (pdf 796 KB)

## Data Availability

No datasets were generated or analysed during the current study.
