# Supplementary material for: Handgrip strength and diameter–product–derived psoas area estimate in end–stage liver cirrhosis: an exploratory single–center analysis
Source: Langenbecks Arch Surg. 2026 Jul 29;411(1):202. doi: 10.1007/s00423-026-04150-y (PMC13421185; doi:10.1007/s00423-026-04150-y)
Supplement: Supplementary file 1 — (pdf 796 KB) [file 423_2026_4150_MOESM1_ESM.pdf]

Online Resources

Online Resource 1

Table S1: Comparison of study parameters by sex

**Parameter**	**Males** ($n = 55$)	**Females** ($n = 41$)	**Mean Diff**	p**-value**
Age (years)	53.5 ± 10.8	48.7 ± 11.7	4.8	0.041
Weight (kg)	84.5 ± 17.0	77.0 ± 16.8	7.5	0.035
Height (cm)	175.9 ± 7.0	165.8 ± 6.5	10.1	<0.001
BMI (kg/m^2)	27.2 ± 5.1	27.7 ± 5.5	−0.5	0.573
MELD score	12.9 ± 4.4	12.8 ± 4.9	0.1	0.895
Handgrip (kg)	47.7 ± 18.9	31.9 ± 17.2	15.8	<0.001
Gait speed (s)	6.3 ± 7.5	11.1 ± 27.4	−4.8	0.146
Total psoas area estimate (mm^2)	3265 ± 911	2228 ± 700	1037	<0.001
Total psoas index (mm^2/m^2)	1075 ± 271	783 ± 220	292	<0.001

Online Resource 2

Table S2: Pearson correlation coefficients for sarcopenia measures[a]

**Variables**	r	p_{adj}**-value**
Handgrip strength correlations		
Total psoas area estimate	0.35	0.002
Total psoas index	0.30	0.008
Gait speed correlations		
Total psoas area estimate	−0.19	0.064[b]
Total psoas index	−0.17	0.100[b]
Additional correlations with the total psoas area estimate		
Weight	0.38	0.002
Height	0.42	<0.001
Age	−0.19	0.064[b]
BMI	0.20	0.054[b]
MELD score	−0.03	0.790[b]

[a] p-values adjusted using Benjamini–Hochberg FDR correction.
[b] Unadjusted p-value (non-significant).

Online Resource 3

Table S3: Comparison of regression models for total psoas area estimate prediction

**Model**	**Variables**	R^2	**SEE**	β	p
Simple linear	Handgrip strength	0.12	935.21	0.35	0.001
Multiple linear		0.27	858.65		
	Weight (kg)			0.25	<0.05
	Height (cm)			0.23	<0.05
	Handgrip (kg)			0.24	<0.05

SEE: Standard error of estimate (mm^2)

Online Resource 4

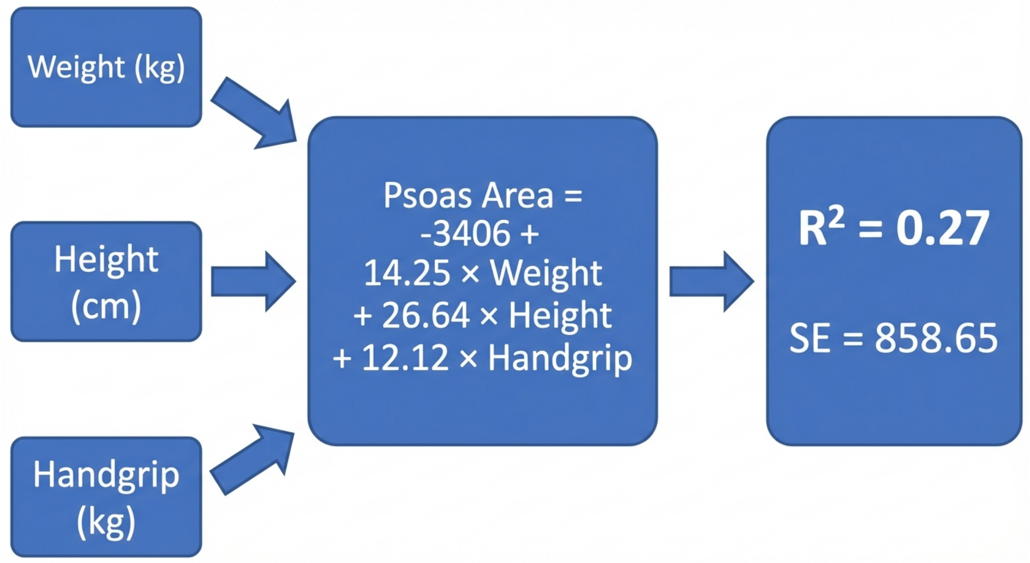

Figure S1: Multiple regression model for psoas area estimate prediction. Schematic showing the prediction model incorporating handgrip strength, weight, and height to estimate the total psoas area ($R^2 = 0.27$, SEE = 858.65 mm^2).

Online Resource 5

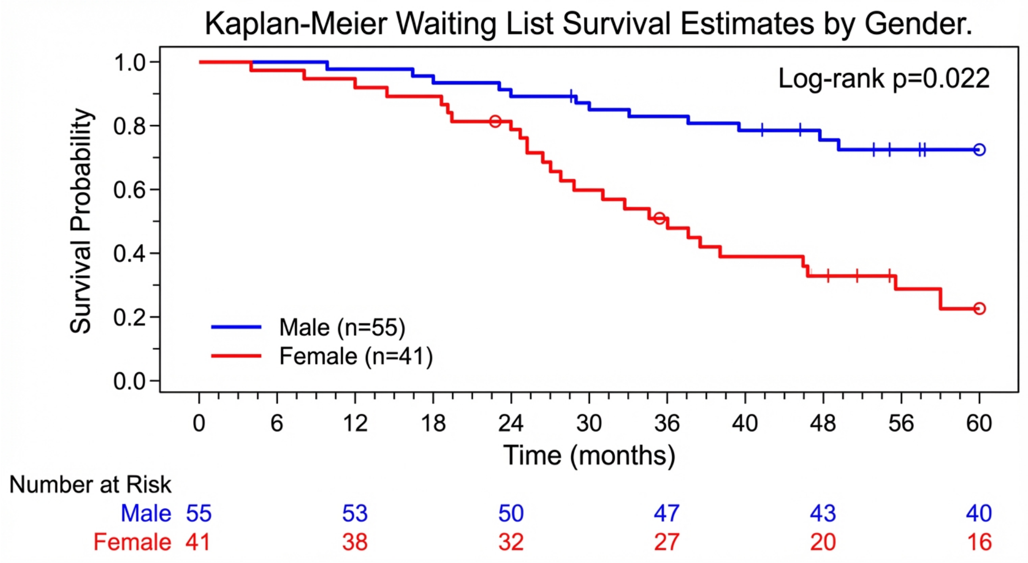

Figure S2: Kaplan–Meier survival curves for waiting list survival by sex. Male patients demonstrated significantly better waiting list survival compared to females (log-rank $p = 0.022$).
